# Association of physiological stress markers at the emergency department to readmission and death within 90 days: a prospective observational study

**DOI:** 10.48101/ujms.v128.9300

**Published:** 2023-05-03

**Authors:** Lee Ti Davidson, Ulf Martin Schilling, Hans J. Arnqvist, Fredrik H. Nystrom, Simona I. Chisalita

**Affiliations:** a Department of Emergency Medicine in Linköping, and Department of Biomedical and Clinical Sciences, Linköping University, Linköping, Sweden; b Department of Urgent Care in Linköping, and Department of Health, Medicine and Caring Sciences, Linköping University, Linköping, Sweden; c Department of Endocrinology in Linköping, and Department of Biomedical and Clinical Sciences, Linköping University, Linköping, Sweden; d Department of Endocrinology in Linköping, and Department of Health, Medicine and Caring Sciences, Linköping University, Linköping, Sweden

**Keywords:** Emergency department, chest pain, shortness of breath, copeptin, MR-proADM, MR-proANP, readmission, multimorbidity

## Abstract

**Background:**

Predicting the risk of readmission or death in patients at the emergency department (ED) is essential in identifying patients who would benefit the most from interventions. We aimed to explore the prognostic value of mid-regional proadrenomedullin (MR-proADM), mid-regional pro-atrial natriuretic peptide (MR-proANP), copeptin, and high-sensitivity troponin T (hs-TnT) to identify patients with a higher risk of readmission and death among patients presenting with chest pain (CP) and/or shortness of breath (SOB) in the ED.

**Methods:**

This single-center prospective observational study included non-critically ill adult patients with a chief complaint of CP and/or SOB who visited the ED at Linköping University Hospital. Baseline data and blood samples were collected, and patients were followed up for 90 days after inclusion. The primary outcome was a composite of readmission and/or death from non-traumatic causes within 90 days of inclusion. Binary logistic regression was used and receiver operating characteristics (ROC) curves were constructed to determine the prognostic performance for predicting readmission and/or death within 90 days.

**Results:**

A total of 313 patients were included and 64 (20.4%) met the primary endpoint. MR-proADM > 0.75 pmol/L (odds ratio [OR]: 2.361 [95% confidence interval [CI]: 1.031 – 5.407], *P* = 0.042) and multimorbidity (OR: 2.647 [95% CI: 1.282 – 5.469], *P* = 0.009) were significantly associated with readmission and/or death within 90 days. MR-proADM increased predictive value in the ROC analysis to age, sex, and multimorbidity (*P* = 0.006).

**Conclusions:**

In non-critically ill patients with CP and/or SOB in the ED, MR-proADM and multimorbidity may be helpful for the prediction of the risk of readmission and/or death within 90 days.

## Introduction

In Sweden, about 15–20% of all hospitalizations are hospital readmissions occurring within 30 days after discharge (1). Readmission is associated with increased morbidity and mortality and related annual costs were estimated to be SEK 2.3 billion (USD 114 million) (2). Preventing avoidable readmissions can profoundly improve the quality of life for patients as well as healthcare’s financial systems. Therefore, hospital readmission and death are considered quality healthcare measures (3).

Identifying high-risk patients at the emergency department (ED) is essential to prevent short- and long-term deterioration. Several scoring systems and triage scales were developed to identify patients at a high risk of catastrophic deterioration in which vital parameters play an important role (4). Identifying patients at high risk but without apparent derangement in vital parameters is tricky. There is no ideal decision-making tool for seemingly non-critical ED patients, and physicians’ assessments of disease severity are inconsistent (5). Furthermore, the prediction of readmission is complex and not all relevant information may be available at the ED, which results in many published tools/scoring systems with often limited predictive value (6). Blood biomarkers can be measured quickly and accurately and are more objective than personal judgment. Biomarkers of physiological stress might help to determine patients at generally increased risk of poor outcomes (7–9) and potentially help identify patients with increased risk of readmission or death after the immediate illness requiring the visit to the ED.

Mid-regional proadrenomedullin (MR-proADM) is the stable portion of the pro-hormone adrenomedullin (ADM), a potent vasodilator peptide expressed in various tissues. It involves fluid-electrolyte homeostasis by acting on the renin-angiotensin-aldosterone system (RAAS) and hypothalamic-pituitary-adrenal axis (10). Mid-regional pro-atrial natriuretic peptide (MR-proANP) is a pro-hormone of the atrial natriuretic peptide (ANP) produced in the cardiac atrium. ANP causes vasodilatation, promotes natriuresis and diuresis, and suppresses the RAAS and sympathetic nervous system (11). Copeptin, a more stable peptide of arginine vasopressin (AVP), is co-secreted from the pituitary gland in equimolar amounts to AVP upon hemodynamic, osmotic, and various other stress-related stimuli (12).

MR-proADM, MR-proANP, and copeptin are surrogate markers reflecting the mature peptides released into the blood circulation. High plasma levels of these prohormones were described in various diseases and found to correlate with disease severity, for example, myocardial infarction, heart failure, respiratory problems, and sepsis (7, 10–15). Elevated plasma levels of MR-proADM, MR-proANP, and copeptin are physiological stress markers, indicating severe underlying disease, which is highly valuable for risk stratification and prognostic information for ED patients (15–18).

In this study, we aimed to evaluate the prognostic value of MR-proADM, MR-proANP, copeptin, high-sensitivity troponin T (hs-TnT), and baseline information available at ED presentation to identify seemingly non-critically ill patients with chest pain (CP) and/or shortness of breath (SOB) at risk of readmission or death within 90 days.

## Material and methods

### Study design

In this single-center prospective observational study, between the years 2013 and 2017, we included a sample of non-critically ill adult patients (18 years old and above) presenting to the ED with CP, SOB, or both, with onset within 7 days (19). The study was performed at Linkoping University Hospital, Sweden, a tertiary care teaching hospital with almost 50,000 annual ED patients. The following were the exclusions criteria:

1. Patients presenting with ST elevated myocardial infarction or a newly discovered left bundle branch block on electrocardiogram (ECG).
2. Critically ill patients (airway compromise, saturation < 90%, respiratory rate > 30 or < 8, heart rate > 120 or < 40 beats per min, systolic blood pressure < 90 mmHg, massive CP, clinically unstable patients for whom urgent medical attention was needed).
3. Patients diagnosed with renal failure with an estimated glomerular filtration rate of less than 20 mL/min/1.73 m².
4. Patients with an advanced focal or spread malignancy.
5. Patients with liver disease or liver failure.
6. Trauma patients.
7. Patients with reduced decision-making ability.

All patients were triaged according to the Rapid Emergency Triage and Treatment System (RETTS©) (Supplementary 1) as described in a previous study (19). Patients’ symptom characteristics and vital parameters, that is, blood pressure, respiratory rate, heart rate, body temperature, peripheral oxygen saturation, and ECG, were collected at triage. The medical history, body weight, and height were recorded. Emergency physicians conducted routine assessments and physical examinations of patients.

Information about baseline comorbidities was collected from the medical records (please refer to Supplementary 2 for a sample of patients’ comorbid conditions in the study).

### Laboratory analysis

Blood samples were collected at ED presentation and frozen at −70°C until analysis. Blood samples for C-reactive protein (CRP), lactate, and hs-TnT were analyzed by clinical practice at Linköping University Hospital’s central laboratory. In cases where the results were unavailable on the first presentation, hs-TnT data were obtained using the analyzed frozen blood sample. Hs-TnT levels analyzed from frozen blood samples correlated well with hs-TnT analyzed at a presentation by the central laboratory (Spearman’s, *r*_s_ = 0.92, *P* < 0.001 [Supplementary 3]).

MR-proADM, MR-proANP, and copeptin were analyzed using a highly sensitive time-resolved amplified cryptate emission technology assay (B.R.A.H.M.S, KRYPTOR, AG, *Hennigsdorf,* Germany). The assay had an analytical detection limit of 0.04 nmol/L, 2.1 and 1.7 pmol/L, while the inter-assay variability was 3.3, 3.0, and 5.2% for MR-proADM, MR-proANP, and copeptin, respectively.

The results of the study markers were unavailable to the attending physician and no intervention was conducted based on these data. The patients’ assessments were left to the discretion of the attending physicians.

### Patient follow-up

Information was collected from the electronic health records (Cambio COSMIC^®^, Linköping, Sweden) used by all regional hospitals, which were integrated with other medical health records and death reports in the state. Follow-up was performed until 90 days after the first ED presentation.

### Main outcome

The primary outcome was a composite of the first unplanned readmission or death within 90 days after study recruitment. Death was defined as death from any non-traumatic cause. Endpoint data were collected from the electronic health records, as described here.

### Ethics

All participants gave their written informed consent. The study was approved by the regional ethical committee of Linköping (diary number 2011/501-31) and conformed to the principles outlined in the Declaration of Helsinki.

### Statistical analysis

Data analyses were conducted using SPSS software (IBM SPSS Statistics, Version 26). A *P*-value (2-tailed) of <0.05 was accepted as statistical significance. Given the exploratory nature of this study, no sample size calculation was performed. The primary outcome was binarily coded; 0 = survivor and no readmission, and 1 = readmission and/or death. Comorbidities were defined as a known medical illness, coded as a binary variable, and dichotomized according to the number of comorbidities: up to two comorbidities (non-multimorbidity group) and more than two comorbidities (multimorbidity group), according to the Swedish National Board of Health and Welfare’s definition (20). The biomarkers MR-proADM, MR-proANP, copeptin, and hs-TnT were dichotomized at a pre-specified cut-off level as in previous studies (19, 21, 22).

Baseline population characteristics were presented for the total cohort and stratified based on the primary outcome. Descriptive statistics were expressed as means and standard deviations or medians with interquartile ranges for continuous variables and as frequencies and percentages for nominal variables. Categorical variables were compared using the chi-square test, while the student’s *t*-test and Mann–Whitney U-test were used to compare continuous variables.

Univariable logistic regression analysis was first performed to identify potential explanatory variables (*P* < 0.05) that could be considered for inclusion in the multivariable binary logistic regression analysis in which a backward elimination was performed. A base model was created, including age and sex as compulsory variables and multimorbidity. MR-proADM, MR-proANP, copeptin, and hs-TnT were added separately to the base model, followed by a combination of studied markers. The adjusted odds ratios (OR) and 95% confidence intervals (CI) were reported as a measure of association.

Receiver operating characteristics (ROC) curves were constructed separately and then compared for the base model; age, sex, and multimorbidity, and for the base model combined with binary MR-proADM > 0.75 nmol/L to determine the prognostic performance with area-under-the-curves (AUCs) for prediction of readmission and/or death within 90 days.

## Results

In total, 313 patients were included in the analysis, of which 64 (20.4%) met the outcome, that is, a composite of readmission and/or death within 90 days.

The population characteristics at inclusion are presented in Table 1. The mean age of the participants was 64 ± 17 years, and 158 (50.5%) were men. Of all included patients, 195 (62.3%) presented with CP only, 61 (19.5%) presented with SOB only, and 57 (18.2%) presented with both complaints. A total of 165 (52.7%) of all patients were admitted for in-hospital care, of whom 58 (35.2%) were admitted for more than 3 days. None of the patients needed intensive care unit (ICU) admission. Within the 90-day follow-up, 61 (19.5%) patients were readmitted, and five (1.6%) deceased, of which two happened at readmission, resulting in the final composite endpoint outcome consisting of 64 (20.4%) patients. A total of 33 (51.6%) of the patients who were readmitted and/or died had multimorbidity. Of the 249 (79.6%) survivors with no readmission events during the 90-day follow-up period, 202 (81.0%) had no more than two comorbidities. The most frequent pre-existing comorbidity was hypertension (47.6%), followed by ischemic heart disease (26.5%) (Supplementary 2). There was no significant difference regarding baseline vital parameters except for lower peripheral oxygen saturation in patients who were readmitted or died within 90 days. At baseline, patients who were either readmitted and died had higher plasma concentrations of CRP, creatinine, lactate, MR-proADM, MR-proANP, copeptin, and hs-TnT (Table 1).

**Table 1:** Baseline characteristic of the study population.

Variables	Overall	Readmission and/or death within 90 days		P
		Yes	No	
Number of patients, *n* (%)	313	64 (20.4)	249 (79.6)	
Male/female, *n* (%)	158 (50.5)/155 (49.5)	37 (57.8)/27 (42.2)	121 (48.6)/128 (51.4)	0.188
Age, years (mean ± SD)	64 ± 17	70 ± 16	63 ± 17	0.003
BMI, kg/m² (mean ± SD)	28.0 ± 5.5	28.6 ± 5.3	27.6 ± 5.6	0.252
Multimorbidity, *n* (%)	80 (25.6)	33 (51.6)	47 (19.0)	<0.001
ACE-I/ARB, *n* (%)	124 (39.9)	37 (58.7)	87 (35.1)	0.001
Diuretic, *n* (%)	76 (24.4)	30 (47.6)	46 (18.5)	<0.001
Chest pain only, *n* (%)	195 (62.3)	32 (50.0)	163 (65.5)	0.023
Shortness of breath only, *n* (%)	61 (19.5)	17 (26.6)	44 (17.6)	0.109
Chest pain and shortness of breath, *n* (%)	57 (18.2)	15 (23.4)	42 (16.9)	0.224
RETTS Blue & Green (No emergency), *n* (%)	6 (2.3)	0	6 (3.0)	0.184
RETTS Yellow (within 120 min), *n* (%)	87 (33.5)	18 (31.0)	69 (34.1)	0.657
RETTS Orange & Red (20 min to urgent), *n* (%)	167 (64.2)	40 (69.0)	127 (62.9)	0.393
Systolic blood pressure (mean ± SD)	147 ± 24	144 ± 23	149 ± 24	0.155
Temperature, °C (mean ± SD)	36.9 ± 0.7	37.0 ± 0.7	36.9 ± 0.6	0.519
Peripheral oxygen saturation, % (mean ± SD)	97 ± 4	95 ± 5	97 ± 3	0.008
Respiratory rate/min (mean ± SD)	19 ± 5	20 ± 6	17 ± 5	0.056
Heart rate/min (mean ± SD)	80 ± 20	82 ± 21	79 ± 19	0.092
Hemoglobin, g/L;(mean ± SD)	139 ± 16	133 ± 19	140 ± 14	0.002
C-reactive protein, CRP, mg/L (median [IQR])	10 (5–10)	10 (5 – 14)	7 (5 – 10)	0.001
Creatinine, μmol/L (mean ± SD)	84 ± 27	98 ± 38	80 ± 23	<0.001
Lactate, mmol/L (mean ± SD)	1.2 ± 0.5	1.3 ± 0.7	1.2 ± 0.5	0.024
Copeptin, pmol/L (median [IQR])	6.2 (3.7 – 13.4)	10.3 (4.2 – 26.6)	5.4 (3.5 – 9.7)	0.044
Hs-TnT, g/L (median [IQR])	9 (6 – 19)	19 (8–33)	7 (4 – 12)	<0.001
MR-proADM, nmol/L (mean ± SD)	0.8 ± 0.5	1.1 ± 0.7	0.7 ± 0.4	<0.001
MR-proANP, pmol/L (median [IQR])	91 (58 – 180)	132 (76 – 283)	79 (53 – 130)	<0.001
Admission, *n* (%)	165 (52.7)	44 (68.8)	121 (48.6)	0.004
Length of hospital stay, > 3 days, *n* (%)	58 (35.2)	22 (34.4)	36 (14.5)	<0.001

BMI: body mass index; IQR: inter quartile range; ACE-I/ARB: angiotensin converting enzyme inhibitors/angiotensin receptors blockers; RETTS: rapid emergency triage and treatment system, color-coded triage with increasing acuity from green to red; Hs-TnT: high-sensitivity troponin T; MR-proADM: midregional pro-adrenomedullin; MR-proANP: midregional pro-A-Type natriuretic.

**Figure 1:**
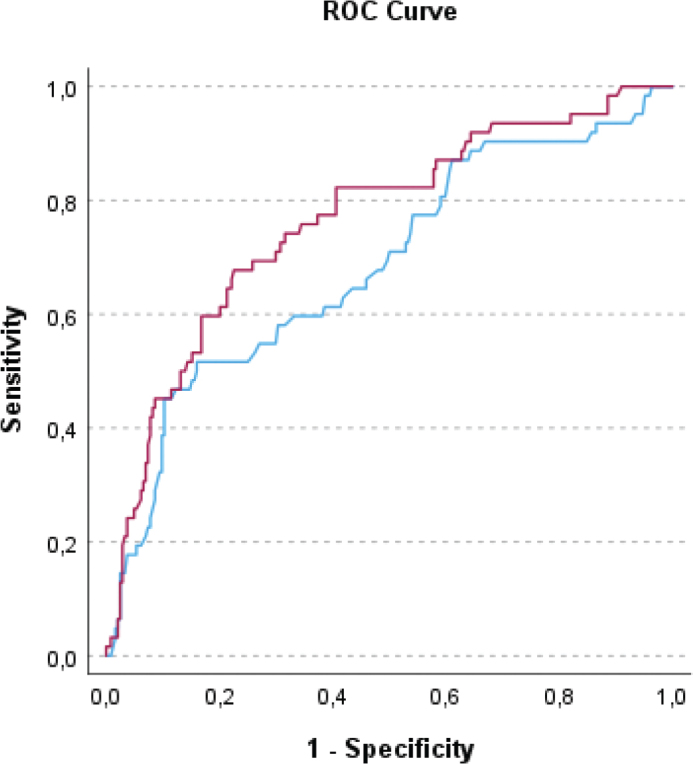
ROC curve for prediction of readmission and/or death within 90 days. Blue = Age + sex + multimorbidity, vs. Red = Age + sex + multimorbidity + MR-proADM > 0.75 pmol/L (P = 0.006)

Peripheral oxygen saturation, multimorbidity, use of angiotensin-converting enzyme inhibitors/angiotensin receptors blockers (ACE-I/ARB), use of diuretics, hemoglobin level, creatinine, lactate, were identified as potential explanatory variables by the univariable binary logistic regression analysis. After adjustment, multimorbidity remained a possible explanatory variable. The result of the multivariable binary logistic regression analyses: a base model with age and sex as compulsory variables, and multimorbidity, added with MR-proADM, MR-proANP, copeptin, and hs-TnT alone or in combination, are presented in Table 2a–f. Hs-TnT was significantly associated with outcome added to age, sex, and multimorbidity (OR: 2.622, 95% CI: 1.255 – 5.471, *P* = 0.010) (Table 2c). However, this significant association was lost when MR-proADM was added to the model (Table 2e). Multimorbidity was the only baseline variable that remained significantly associated with readmission and/or death within 90 days; *P* = 0.009. Patients with multimorbidity had an OR of 2.6 (95% CI: 1.282 – 5.469) of being readmitted or dying within 90 days compared with those with fewer than two or no comorbidity (Table 2a–f). MR-proADM > 0.75 pmol/L was the only stress marker that remained significantly associated with readmission and/or death within 90 days (OR: 2.4, 95% CI: 1.031 – 5.407, *P* = 0.042) (Table 2e–f). In ROC analysis, the base predictive model consisting of age, sex, and multimorbidity showed an AUC of 0.686 (95% CI: 0.607 – 0.764), and the addition of MR-proADM to the base model showed an AUC of 0.764 (95% CI: 0.695 – 0.833), see Figure 1. Adding MR-proADM had a significantly increased predictive value compared with the base predictive model, that is, age, sex, and multimorbidity, separately (AUC difference 0.078, 95% CI: 0.022 – 0.134, *P* = 0.006).

**Table 2:** Multivariable binary logistic analysis for association of age, sex, and multimorbidity with MR-proADM, MR-proANP, hs-TnT, and copeptin, respectively, and in combination, to readmission and/or death within 90 days.

Variables in predicting models	OR (95% CI)	P
2a Age	0.996 (0.974 – 1.019)	0.752
Sex	1.478 (0.807 – 2.707)	0.206
Multimorbidity	3.184 (1.600 – 6.337)	<0.001**
MR-proADM > 0.75 pmol/L	2.514 (1.235 – 6.118)	0.011*
2b Age	0.995 (0.969 – 1.021)	0.690
Sex	1.443 (0.791 – 2.633)	0.232
Multimorbidity	3.653 (1.857 – 7.186)	<0.001**
MR-proANP > 120 nmol/L	2.009 (0.897 – 4.500)	0.090
2c Age	0.996 (0.973 – 1.020)	0.743
Sex	1.262 (0.687 – 2.321)	0.453
Multimorbidity	3.156 (1.593 – 6.252)	<0.001**
Hs-TnT > 14 g/L	2.622 (1.255 – 5.471)	0.010*
2d Age	1.013 (0.990 – 1.036)	0.283
Sex	1.463 (0.790 – 2.709)	0.226
Multimorbidity	3.361 (1.699 – 6.646)	<0.001**
Copeptin > 10 nmol/L	0.979 (0.492 – 1.951)	0.952
2e Age	0.989 (0.966 – 1.014)	0.390
Sex	1.380 (0.738 – 2.579)	0.313
Multimorbidity	2.878 (1.427 – 5.802)	0.003*
MR-proADM > 0.75 pmol/L	2.153 (1.009 – 4.592)	0.047*
Hs-TnT > 14 g/L	1.859 (0.846 – 4.088)	0.123
2f Age	0.991 (0.964 – 1.019)	0.527
Sex	1.657 (0.855 – 3.209)	0.134
Multimorbidity	2.647 (1.282 – 5.469)	0.009*
MR-proADM > 0.75 pmol/L	2.361 (1.031 – 5.407)	0.042*
MR-proANP >120 nmol/L	1.224 (0.490 – 3.061)	0.665
Hs-TnT > 14 g/L	2.244 (0.920 – 5.476)	0.076
Copeptin > 10 nmol/L	0.476 (0.211 – 1.075)	0.074

OR: odds ratio; CI: confidence interval; MR-proADM: midregional pro-adrenomedullin; MR-proANP: midregional pro-A-Type natriuretic; Hs-TnT: high-sensitivity troponin T.

## Discussion

This study found that MR-proADM and multimorbidity were related to readmission and/or death in non-critically ill patients with CP and/or SOB 90 days after ED presentation. There was no significant difference regarding baseline vital parameters except for lower peripheral oxygen saturation in patients who were readmitted or died within 90 days. The baseline plasma concentrations of hemoglobin, creatinine, CRP, MR-proANP, hs-TnT, and copeptin were higher but not independently associated with the risk of readmission and/or death within the 90-day follow-up period.

Our findings for MR-proADM were consistent with those of earlier studies that evaluated the utility of prognostic markers in patients at the ED. In selected, potentially critically ill populations, MR-proADM was identified as a stress marker with the ability to predict adverse coronary events, clinical deterioration after ED admission, readmission, and all-cause mortality, and as a triage biomarker for hospitalization reduction (8, 18, 23–25). MR-proADM was considered a better predictor of poor outcomes independent of other biomarkers combined (MR-proANP, copeptin, hs-TnT, N-terminal pro-B-type natriuretic peptide, C-terminal pro-endothelin-I, or CRP) (7, 9, 21, 23, 25, 26). Additionally, MR-proADM assessment could accurately identify disease progression in patients with infection at the ED and safely increase outpatient management without increasing the number of readmissions or death (25). However, most of these studies were conducted in selected ED patients with specific diseases or age groups. Our study supports earlier results and shows that MR-proADM may be able to predict poor outcomes even in unselected, non-critically ill ED patients at the earliest point of care.

In our study, having more than two medical diagnoses defining multimorbidity was the only available baseline information in the ED significantly associated with readmission and/or death within 90 days. The number and disease conditions included in multimorbidity studies vary (27). At the same time, it is accepted that the cumulative effects of illnesses are compounded by the addition of further comorbidities with increasing levels of disability and utilization of healthcare resources (28). Multimorbidity increases the risk of hospital admission, more extended hospital stays, readmission, and mortality (29). The mere addition of a disease condition is a simplified method to provide a proxy for identifying frailty, chronic disease burden, and functional status.

In contrast to the immediate predictive value of copeptin in critically ill patients (30), we found that copeptin had no predictive value for readmission and/or death within 90 days in this non-critically ill patient population. This was in line with previous studies showing that copeptin had no added value for prognostic information over that provided by troponin or MR-proADM (26, 31). A study conducted in a population of critically ill patients admitted to the ICU found that the primary determinant of elevated copeptin was the severity of critical illness, as reflected by organ failure and hemodynamic alterations, not the underlying etiologic (17). Our study focused on a population of non-critically ill patients that may have contributed to copeptin’s negative result.

Hs-TnT is known as an organ-specific marker used to diagnose myocardial infarction. Studies showed that a hs-TnT level above the 99th percentile was common in ED patients with CP but with no myocardial infarction (32) and chronic myocardial injury defined as stable hs-TnT levels > 14 ng/L was a strong marker for death and cardiovascular events (33). CRP and lactate were important in early triage, and previous studies showed their predictive effect on readmission and adverse events (34–37). However, in our population, neither vital signs nor established markers CRP, lactate, nor hs-TnT were significantly associated with readmission and/or death within 90 days. By our findings, the prognostic value of vital parameters, CRP, lactate, and hs-TnT in non-critically ill patients seems beneficial mainly in the short term.

Age and gender are important predictors of various prognostic models for diseases, readmission, and death (38–40). A study population with heart failure showed that women had a 2.5 times greater risk for rehospitalization within 90 days than men (39). At the same time, another study from the population-based longitudinal study Swedish Adoption/Twin Study of Ageing (SATSA) showed that higher age and male sex increase the risk of readmission (38). In our study, including adults 18 years and above, the mean age was higher in patients who were readmitted and/or died within 90 days. However, in our non-critically ill ED population with undifferentiated CP and/or SOB, neither age nor gender had a significant predictive value for readmission and/or death within 90 days after adjustment for multimorbidity, MR-proADM, MR-proANP, hs-TnT, and copeptin.

Most readmission risk prediction models have a poor predictive ability (6) and focus on specific conditions or ages (41). Many are based on data available only after discharge, requiring burdensome data collection beyond that readily available from routine clinical care records (42), rendering their application difficult, if possible, in the immediate emergency visit. Cumulative comorbidities, that is, multimorbidity, and MR-proADM, are potential variables that can be used early in the patient’s pathway to identify patients at risk of readmission and/or death within 90 days.

The strengths of our study include its prospective nature and real-life ED patients with various underlying diseases. We used the cumulative number of comorbidities as a proxy for morbidity burden to simplify ED decisions, making it as non-cumbersome as possible for patients’ early care. To our knowledge, this is the first study of the association between stress biomarkers and unplanned readmission and/or death within 90 days among non-critically ill ED patients. We excluded easily identifiable critically ill patients, those with pronounced SOB, massive CP, or ST-segment elevation confirming myocardial infarction, and clinically unstable patients for whom urgent medical attention was needed. This was deliberate considering the difficulties in the identification and risk stratification of critically ill patients without apparent derangement in the vital parameters. This is a significant difference in patient selection compared with previous blood marker studies conducted in the ED (7–9, 23).

Our study had limitations. We used specific cut-offs, which might decrease the analytical power compared with continuous analysis. However, we used known cut-off values as in the previous study, which makes it easier for application in daily clinical practices. Given the exploratory nature of this study, no sample size estimation was performed, and no causal relationship could be determined. As this was a single-center study, the results may not apply to other settings.

## Conclusions

In non-critical patients presenting with undifferentiated CP and/or SOB in the ED, multimorbidity is the only baseline information of importance for predicting readmission and/or death within 90 days. MR-proADM may be a helpful biomarker for identifying ED patients with CP and/or SOB at higher risk of readmission and/or death within 90 days. MR-proADM might facilitate ED discharge decisions and increase safe outpatient management. Considering our findings, further studies to determine the prognostic value of MR-proADM in the unselected non-critically ill ED population seem warranted.
