# Supplementary material for: Association of physiological stress markers at the emergency department to readmission and death within 90 days: a prospective observational study
Source: Ups J Med Sci. 2023 May 3;128:10.48101/ujms.v128.9300. doi: 10.48101/ujms.v128.9300 (PMC10202081; doi:10.48101/ujms.v128.9300)

Supporting information/supplementary materials

Supplementary 1: **The national Swedish triage system: Rapid Emergency Triage and Treatment System (RETTS©) scores. Explanation of how the triage of patients was performed at the study site emergency department.**

Rapid emergency triage and treatment system (RETTS©) scores (https://predicare.eu/), the predominant triage system in Sweden today, assign patients to a five-level color-coded triage scale based on cut-off levels for vital signs and 59 chief complaint-based algorithms called ESS (emergency symptoms and signs). Each ESS included one or more chief complaints classified according to the International Statistical Classification of Diseases and Related Health Problems 10th Revision for 2007 (ICD-10). The RETTS© provides recommendations for decisions according to local facilities and does not frame the maximum waiting time. In our setting, the local guidelines recommended the maximum timeframe for the triage level as follows: triage level red = urgent assessment, triage level orange = assessment within 20 minutes, triage level yellow = within 120 minutes, and triage level green or blue = no immediate emergency assessment. In our setting, patients with CP and SOB were usually assigned higher risk categories based on symptoms and nurses' gestalt, despite normal vital signs. An example of a triaging algorithm for chief complaint chest pain, according to RETTS©, can be accessed from Wireklint et al. https://doi.org/10.1016/j.ienj.2017.12.003.

Supplementary 2: **Pre-existing known comorbidity among patients on study inclusion**

Comorbidity	N (%) of the total population, N=313
Hypertension	149 (47.6)
Ischemic Heart Disease	83 (26.5)
Diabetes Mellitus	52 (16.6)
Atrial Fibrillation	52 (16.6)
Heart Failure	38 (12.1)
Asthma	34 (10.9)
Systemic Inflammatory Disease	29 (9.3)
Previous Cerebrovascular Accident	27 (8.6)
Chronic Obstructive Pulmonary Disease	25 (8.0)
Previous Thromboembolism	20 (6.4)
Renal Disease	17 (5.4)
Nonadvanced Malignancy	15 (4.8)

Supplementary 3: **Scatter plot of hs-TnT analyzed at first presentation compared to frozen blood samples obtained by the central laboratory.**

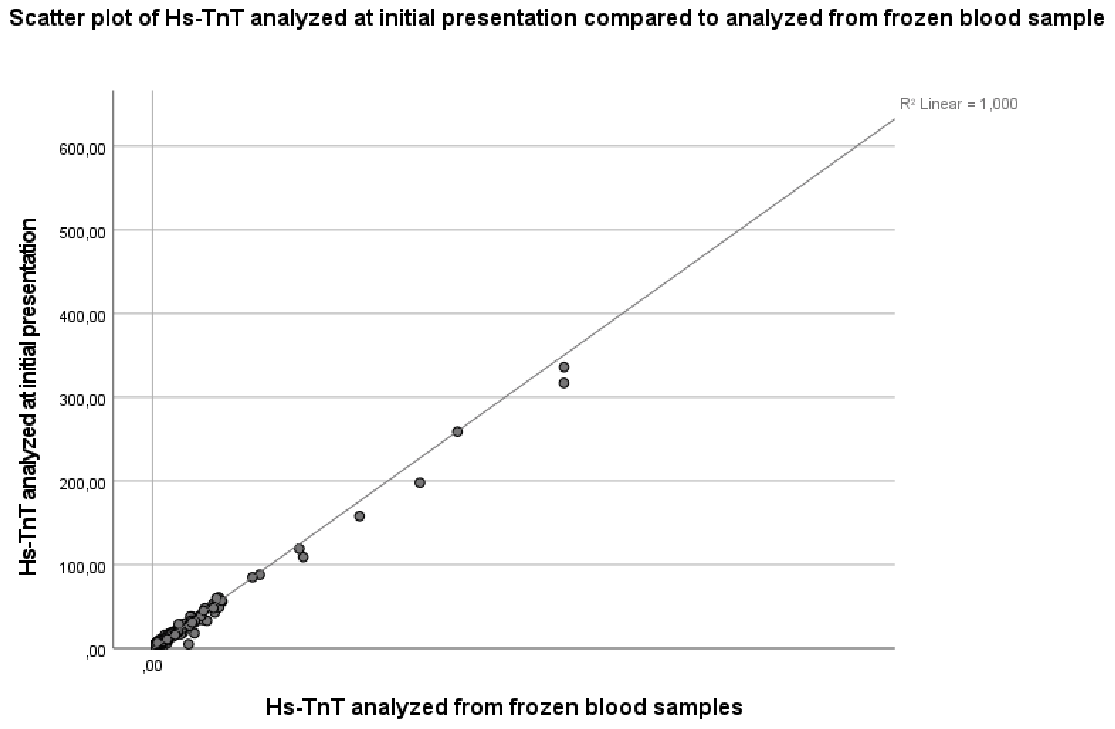

Supplement: Supplementary file 1 [file UJMS-128-9300-s001.pdf]
